# The gut pathobiome: a new frontier in the management of the critically ill

**DOI:** 10.62838/jccm-2026-0001

**Published:** 2026-04-30

**Authors:** Krisztina Madách

**Affiliations:** Department of Anesthesiology and Intensive Therapy, Semmelweis University, Budapest, Hungary

**Keywords:** pathobiome, dysbiosis, gastrointesinal microbiota, multi-organ dysfunction syndrome, gut-organ axis, post-intensive care syndrome

The management of critically ill patients has undergone a radical transformation over the last decades. We have refined the art of organ support and replacement therapies, yet, despite these technological leaps, multi-organ failure remains a primary driver of mortality in the Intensive Care Unit (ICU). Furthermore, as short-term survival rates improve, we are increasingly confronted with the substantial burden of long-term morbidity. While scoring systems like the Sequential Organ Failure Assessment (SOFA) frequently overlook the gut due to the lack of easily quantifiable biomarkers, recent scientific evidence increasingly highlights its pivotal importance. The mapping of the human microbiome has revealed that the gastrointestinal tract is not merely a component of multi-organ dysfunction syndrome but a central orchestrator of it. The gut microbiota engages in complex crosstalk with distal organ sites, effectively shaping the clinical trajectory of critical illness both in the acute phase and throughout long-term recovery [1].

The healthy human microbiota is a diverse ecosystem essential for systemic homeostasis, regulating metabolic processes, immune maturation, and systemic immunomodulation. By maintaining intestinal barrier integrity, the microbial community serves as a vital protective shield for the host. This symbiotic relationship is defined by diversity, which acts as colonization resistance against opportunistic pathogens [2].

The onset of critical illness is an ecological cataclysm from the aspect of the microbiota. Within hours of ICU admission, the intestinal landscape shifts toward a “pathobiome”— a state where diverse communities are replaced by highly virulent, often multidrug-resistant (MDR) organisms. In this state, bacteria sense the host’s stress signals — such as catecholamines and opioids — and respond by activating virulence genes that increase their invasiveness and inflammatory potential. Recent metagenomic studies have shown that this collapse in diversity is directly associated with increased susceptibility to secondary infections and higher mortality rates [3, 4].

To manage the pathobiome, we must acknowledge how our life-saving interventions contribute to its formation. Antibiotics are the most significant factors, often wiping out the bacteria that produce protective metabolites [5]. Beyond antibiotics, the "standard of care" frequently works against microbial health. Proton pump inhibitors alter the pH of the digestive tract, facilitating the migration of oropharyngeal bacteria to the lower gut and increasing the risk of nosocomial pneumonia [6]. Opioids induce stasis, preventing the natural cleansing of the bowel and promoting bacterial overgrowth, while also directly activating bacterial virulence via inter-kingdom signaling (the direct communication between host neuroendocrine factors and bacterial receptors) [7]. Furthermore, the absence of early enteral nutrition deprives the microbiome of the fiber needed to maintain the mucosal barrier and produce protective mucin [8]. Finally, the use of high dose vasopressors, while may be essential for hemodynamic stability, can exacerbate mesenteric ischemia, further compromising the intestinal niche and promoting the dominance of the pathobiome [9]. The intense selective pressure of the ICU environment facilitates the dominance of multidrug-resistant (MDR) strains, further complicating therapeutic interventions and fueling systemic inflammation.

The impact of the pathobiome extends far beyond the intestinal lumen, influencing remote organs through a network of complex gut-organ axes. Beyond the well-established gut-lung and gut-brain axes - where microbial products prime alveolar macrophages for respiratory failure and neurotoxic metabolites breach the blood-brain barrier to drive ICU delirium - this systemic influence encompasses the entire host physiology. Dysbiosis-induced systemic inflammation and toxemia significantly impair skeletal muscle homeostasis, contributing to ICU-acquired weakness and muscle wasting. The translocation of pathogen-associated molecular patterns (PAMPs) via the portal circulation and mesenteric lymph exacerbates hepatic inflammation and triggers microvascular dysfunction in the kidneys, reinforcing the cycle of acute kidney injury and liver dysfunction. This integrated crosstalk confirms that the pathobiome acts as a systemic driver of multi-organ dysfunction, affecting metabolic, structural, and cognitive recovery [10].

Despite the clinical promise of microbiome analysis, significant methodological hurdles remain that prevent its routine use at the bedside. Current sequencing technologies often fail to provide the real-time, high-resolution data required for acute clinical decision-making. A major limitation is the inability of standard 16S rRNA sequencing to distinguish between viable, metabolically active bacteria and dead microbial DNA fragments, which can result in a misleading representation of the functional pathobiome. Furthermore, the lack of standardized protocols for fecal sample collection and DNA extraction across different ICUs creates significant variability in results, hindering multicenter comparisons. A single "snapshot" sample upon admission is hardly sufficient to capture the rapid shifts of the microbiome during the dynamic course of critical illness, necessitating a move toward high-frequency longitudinal monitoring integrated with multi-omics approaches to truly enable precision medicine [11].

Addressing the pathobiome requires 'microbiome-conscious' critical care, prioritizing antibiotic stewardship and early enteral nutrition, while strictly limiting therapies known to drive dysbiosis, such as the indiscriminate use of proton pump inhibitors, unmonitored opioid sedation, and excessive vasopressor support. While Selective Decontamination of the Digestive Tract (SDD) remains a controversial preventative strategy, recent large-scale trials continue to debate its impact on long-term resistance patterns [12]. The future likely lies in microbiota restoration. Fecal Microbiota Transplantation (FMT) is being tested for MDR organism eradication in the ICU setting [13], while synbiotics and next-generation probiotics show promise in reducing septic complications by restoring epithelial integrity [14].

The transition from a passive victim to an active driver of multi-organ dysfunction identifies the gut pathobiome as a potential therapeutic target in the ICU. Microbiome preservation is no longer elective; it is an essential strategy for mitigating systemic inflammation and reducing sepsis-related mortality while simultaneously addressing the long-term sequelae of post-intensive care syndrome, such as cognitive decline and physical frailty. As we move toward an era of precision medicine, integrating real-time microbial monitoring and targeted ecological restoration represents a promising frontier for the next generation of critical care.
